# Effect of exercise interventions based on family management or self-management on glycaemic control in patients with type 2 diabetes mellitus: a systematic review and meta-analysis

**DOI:** 10.1186/s13098-023-01209-4

**Published:** 2023-11-14

**Authors:** Chenyang Dong, Ruoya Liu, Zhiyang Huang, Yang Yang, Shiyuan Sun, Ran Li

**Affiliations:** School of Sport Science, Beijing Sport University, 48 Xinxi Road, Haidian District, Beijing, China

**Keywords:** Type 2 diabetes mellitus, Exercise interventions, Self- management, Family management, Glycaemic control, Meta-analysis

## Abstract

**Background:**

Most studies about exercise interventions for patients with type 2 diabetes mellitus (T2DM) have been conducted in hospitals or labs, but it is unclear whether study findings from this specific condition can be generalised to real-life T2DM communities. If patients with T2DM can exercise on their own or with family members, it may also reduce the need for patient supervision by medical staff, thereby reducing the burden of medical treatment and improving condition management's cost-effectiveness and practicability. Much of the current research on exercise interventions for T2DM was focused on the type of exercise and less on the mode of management, so we aimed to examine the effect of exercise interventions based on family management or self-management on glycaemic control in patients with T2DM.

**Methods:**

Articles were searched from eight Chinese and English databases. Randomized control trials (RCTs) published in English and Chinese, from inception to October 17, 2022, were included in this review. The methodological quality of the included studies was assessed using the RCT risk of bias assessment tool provided by the Cochrane Collaboration’s tool. Meta-analysis was performed using Rev Man 5.4 and Stata 15.0 software. Heterogeneity was investigated using sensitivity analysis and subgroup analyses. Publication bias was tested by funnel plot and Egger's asymmetry tests.

**Results:**

A total of 10 studies with a total of 913 subjects were finally included in this review. The Meta-analysis showed that exercise interventions based on family management or self-management were more effective than control groups in reducing HbA1c (Z = 3.90; 95% *CI* *MD* = − 0.81; − 1.21 to − 0.40; *P* < 0.0001), fasting glucose (Z = 4.63; 95% CI *MD* = − 1.17; − 1.67 to − 0.68; *P* < 0.00001), 2-h plasma glucose (Z = 5.53; 95% CI *MD* = − 1.84; − 2.50 to − 1.19; *P* < 0.00001), and Low-density lipoproteins levels (Z = 3.73; 95% *CI* *MD* = − 0.38; − 0.58 to − 0.18; *P* = 0.0002).

**Conclusions:**

Exercise interventions based on family management or self-management can significantly reduce glycosylated haemoglobin, fasting glucose, 2-h plasma glucose, and Low-density lipoproteins levels in patients with T2DM, which can effectively delay disease progression and reduce the risk of developing complications. In the future, for exercise interventions based on family or self-management, this review recommended that exercise intervention programmes should be formulated according to 30–60 min per session, more than three times per week, for more than six months of aerobic exercise or aerobic combined with resistance exercise.

**Supplementary Information:**

The online version contains supplementary material available at 10.1186/s13098-023-01209-4.

## Introduction

Type 2 diabetes mellitus (T2DM) is a prevalent endocrine metabolic condition [1] characterized by impaired insulin sensitivity of peripheral tissue such as the hepatic, muscle, and fatty tissue [2]. By 2021, there were an estimated 537 million people with diabetes worldwide, and in 2045, this number is projected to rise to 783 million [3]. Of these, T2DM accounted for more than 90% [3]. It has imposed a significant economic burden on individuals and society.

A number of studies have confirmed that scientific and rational exercise has a positive effect on the treatment of T2DM [4–6]. The latest exercise guidelines from the American Diabetes Association [7] recommended that adults with T2DM should perform moderate-intensity aerobic exercise at least three times per week for a total duration of 150–300 min, and resistance exercise two to three times per week. Regular aerobic exercise training could improve blood glucose and reduce HbA1c by 0.5–0.7% in adults with T2DM [8–11]. Resistance exercise training in older adults with T2DM improved lipids by 10–15% [12] and reduced HbA1c threefold [13]. A meta-analysis[11] have shown that aerobic exercise, resistance exercise, and aerobic combined resistance exercise all have beneficial effects on blood glucose and insulin sensitivity.

Most studies about exercise interventions for patients with T2DM that have taken place were mostly focused on hospitals or laboratories [14], but it is unclear whether study findings from this specific condition can be generalised to real-life T2DM communities. Moreover, some studies [15, 16] found that patients with T2DM had low adherence to exercise, which led to unsatisfactory management of the disease. The study by Umeh et al. [15] investigated whether patients with T2DM engaged in regular physical activity, i.e., whether they exercise at a certain frequency for a certain period of time per week, to determine their adherence to exercise. Their findings showed that 40.7% of T2DM patients had poor adherence to exercise, and 79.63% of participants who did not adhere to exercise had worse glycaemic control. T2DM is a complex disease that can lead to multiple complications [17], and the patients require long-term independent exercise to effectively manage their disease. Some research [18, 19] has shown that self-management or family accompaniment can improve the adherence of patients with T2DM and help them to manage and improve their disease better. Family or self-management may be a potential strategy for exercise intervention management in T2DM.

Currently, more and more studies about family or self-management of T2DM have involved exercise interventions [20–29]. However, these exercise interventions had various exercise intervention programmes and outcomes, which led to limitations such as the lack of evidence for specific exercise modalities in similar exercise intervention studies. In addition, To solve these deficiencies, this study hypothesised that the meta-analysis of relevant studies would (1) have assessed the impact of exercise interventions based on family or self-management on glycaemic control in T2DM, which could understand the feasibility of the management of this exercise intervention in real-life situations, and (2) have provided recommendations for the detailed management style of family or self-management, as well as for the specific modes of exercise interventions under this management style, thus providing evidential support for guidelines on management and exercise interventions in T2DM.

## Materials and methods

### Protocol and registration

This review was performed according to the Preferred Reporting Items for Systematic Reviews and Meta-Analyses (PRISMA) checklist [30]. The protocol for this systematic review was registered on PROSPERO (registration number: CRD42023392011). The RRISMA checklist is listed in Additional file 2.

### Search strategy

The pre-search was conducted to identify the final search terms. Subsequently, databases from PubMed, EMBASE, Web of Science, The Cochrane Library, China National Knowledge Infrastructure, Weipu Database for Chinese Technical Periodicals, CBM, and Wanfang Database will be searched for pertinent research. The search strategy used a mix of Mesh and free text terms and was determined after repeated pre-searching. Consequently, search terms related to “type 2 diabetes”, “exercise”, and “community” were used. The search was limited to studies published in English or Chinese from the origin to October 17, 2022. The specific search strategies used in four English databases are listed in Additional file 1.

### Inclusion and exclusion criteria

Inclusion criteria: (i) Patient: All patients must be adults (18 years or older) with T2DM, which is characterized by a fasting blood glucose level of 7.0 mmol/L (126 mg/dL), a 2-h plasma glucose level of 11.1, or a glycated haemoglobin (HbA1c) of less than 6.5% [31]. (ii) Intervention: Exercise interventions are delivered in the community more than 50% of the time and usually involve a dedicated exercise intervention programme (frequency; intensity; duration; type) (iii) Comparison: Comparison group or control group (conventional treatment or non-exercise interventions). (iv) Outcomes: HbA1c (v) Management: self-management: exercise by themselves at home or any autonomous form of exercise after a prescription or exercise programme has been originally provided to the patient by a doctor; family management: Exercise with family members (vi) Type of Research: RCTs.

Exclusion criteria: (i) People with acute and chronic diseases that are not suitable for sports, patients with serious complications or other serious diseases, women with gestational diabetes, and people with impaired glucose tolerance (ii) Interventions only mention exercise without a specific exercise program (iii) Repeat studies or sub-studies already included in the trial (iv) Conference Reports (v) There is no abstract in the literature.

### Study selection

The literature retrieved in the database was uniformly imported into Endnote X9 for further screening. Two researchers (Chenyang Dong and Zhiyang Huang) independently selected the retrieved literature for the title, abstract, and full text based on inclusion and exclusion criteria. The differences were resolved by a third researcher (Ran Li) when two researchers had different opinions.

### Data extraction

The data were extracted independently by two researchers (Chenyang Dong and Ruoya Liu) using a uniformly designed form. Data information included basic information of the literature, i.e., first author, year of publication, country, sample size (intervention/control group), mode of administration, outcomes, results, adverse events, etc.; interventions, such as type of exercise, duration, frequency, etc.; primary outcomes: HbA1c; secondary outcomes: fasting blood glucose, 2-h plasma glucose, BMI, blood pressure, lipids, etc. In addition, we also extracted descriptive statistics such as the number of study participants, mean, and standard deviation of outcome measures for the intervention and control groups. If we encountered differences in the extraction process, we discussed or referred to a third researcher (Ran Li) to decide. We placed baseline data for all outcomes in Additional file 3.

### Quality appraisal

As the included articles were randomized controlled trials, methodological quality was assessed with the risk of bias assessment tool provided by the Cochrane Collaboration’s tool [32]. Two researchers (Chenyang Dong and Yang Yang) independently assessed each of the 7 aspects of random sequence generation (selection bias), allocation concealment (selection bias), blinding of researchers and subjects (performance bias), blinding of outcome testers (detection bias), incomplete outcome data (attrition bias), selective reporting (reporting bias), and other biases. The evaluation results were categorized into 3 grades: low, unclear, and high risk of bias. A "Summary of Findings Table" created with GRADEpro GDT (Evidence Prime Inc., McMaster University, 2020) was used to summarize the overall quality of the evidence by two researchers. Any inconsistencies found during the assessment process were discussed by inviting a third reviewer (Ran Li) and addressed by consensus.

### Data analysis

Meta-analyses were performed using Rev Man 5.4 and Stata 15.0 software. The weighted mean difference (*WMD*) was chosen to determine the effect of the intervention group on the outcomes compared to the control group because all of the data included in this study were continuous variables. The weighted mean difference, which is the difference between the two means, can be calculated as follows: *WMD* = ‾X_1 −_‾X_2._ The weight of each original study's mean difference (e.g. the size of each study's effect on the meta-analysis merged statistic) was determined by the precision of its effect estimate. The weight given to each study is chosen to be the inverse of the variance of the effect estimate in Rev Man (i.e. one over the square of its standard error)[33].Blood glucose (except HbA1c) and lipids were both measured in mmol/L. HbA1c was measured in %, BMI was measured in kg/m^2^ and blood pressure was measured in mmHg. If the units did not match during the data analysis, the conversion was done first. The unit conversion between mg/dl and mmol/l: blood glucose, 1 mmol/L = 18 mg/dL; HDL, 1 mmol/L = 38.66 mg/dL; LDL, 1 mmol/L = 38.66 mg/dL; TG, 1 mmol/L = 88.6 mg/dL. The overall effect was tested by a z-test with 95% confidence interval (*CI*). The *I*^2^ statistic and Q test were used to assess the heterogeneity of the included studies. Two methods of analysis are available in RevMan for meta-analysis of continuous data: the inverse variance fixed-effect method and the inverse-variance random-effects method [33]. When heterogeneity was present (*I*^2^ ≥ 50% or *P* ≤ 0.10), a random-effects model was used, and vice versa (*I*^2^ < 50% and *P* > 0.10), a fixed-effects model was used [33]. In order to identify the causes of heterogeneity, subgroup analysis was utilized. Pre-determined subgroups included frequency of intervention (3 times/week, > 3 times/week), types of exercise (aerobic exercise; aerobic combined resistance exercise), duration of intervention (short- to medium-term: 3–6 months; long-term: > 6 months), and baseline levels of HbA1c (≤ 7.5%, > 7.5%) and fasting blood glucose (≤ 7.5 mmol/L, > 7.5 mmol/L). The sensitivity analysis was performed by deleting each study one at a time to identify the source of heterogeneity and to evaluate the reliability of the meta-analysis results. Publication bias was tested by funnel plot and Egger's asymmetry tests. Potential publication bias was indicated by a significant statistical test (*P* < 0.05). If publication bias was present, the trim and fill method was used for adjustment.

## Results

### Search results

**Fig. 1:**
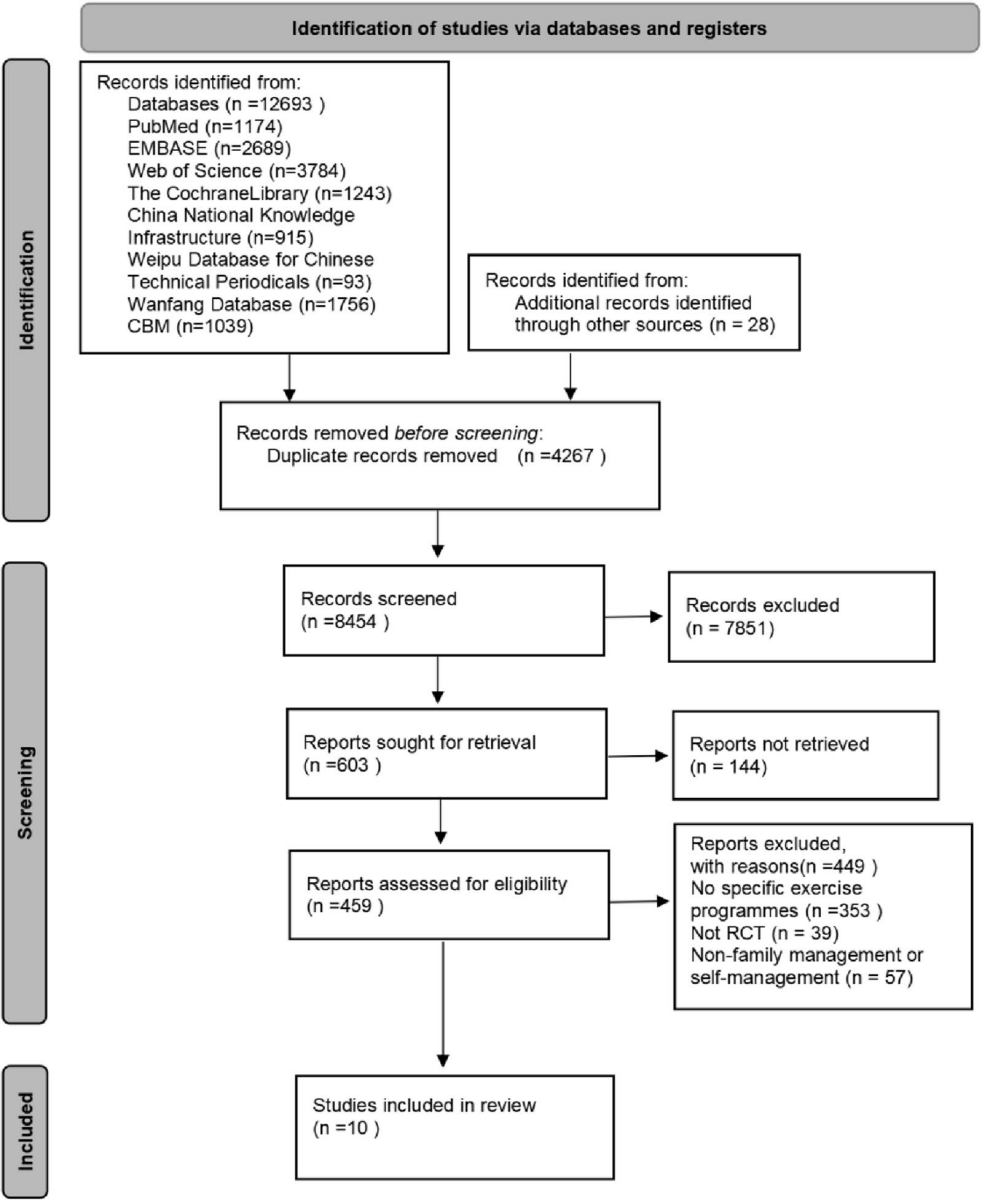
PRISMA flowchart

A total of 12,693 articles were provided by electronic searches, and 28 articles were obtained through other sources. Based on titles, abstracts, or duplicated articles, the 12090 of them were excluded. Eventually, 10 articles [20–29] were included for meta-analysis. The specific screening process for articles is shown in Fig. 1.

### Characteristics of the included studies

All included studies were published between 2004 and 2022. There were 913 patients in the included articles, containing 463 patients in the intervention groups and 450 patients in the control groups. Patients in 8 articles exercised at home by themselves; patients in 2 articles exercised with family members. The duration of the intervention ranged from 3 to 12 months. Nine articles reported on fasting glucose or HbA1c; five articles reported on BMI or blood pressure; four articles reported on Low-density lipoproteins and triglycerides; and three articles reported on 2-h postprandial glucose. The specific basic characteristics are summarized in Table 1.

**Table 1:** Basic characteristics of the included studies

Author (Year)/Country	Study Design	Sample Size, n(Intervention/Control)	Age	Intervention (Type, Duration, Frequency)	Comparison	Management style	Outcomes
Alessandra et al. [20] (2022)/USA	RCT	I:50 C:50	I: 72.3 ± 4.0 C:71.4 ± 3.7	**Type:** CT **Duration:**60 min **Frequency:**3times/week for12 months	No external weight loss or exercise programs	**Self-management:** participants transitioned their regular exercises to community-fitness centers of their choice and homes	HbA1c, FBG, 2hPG
Aifan Chen et al.[21] (2021)/ China	RCT	I:68 C:59	I:72.29 ± 4.54 C:71.71 ± 6.08	**Type:** AT **Duration:**30 ~ 60 min **Frequency:** ≥ 5 times/week for 12 months **Intensity:** Medium intensity	routine treatment	**Self-management:** Patients were exercising autonomously with exercise management and monitoring of blood glucose	FBG, SBP, DBP
Xiaoli Wang et al. [22](2019)/ China	RCT	I:47 C:50	I:61.76 ± 10.54 C:61.67 ± 10.51	**Type:** Qigong、BaDuanJin **Duration:**30 min **Frequency:** ≥ 5 times/week for 12 months	Treatment with original medication without any prescribed exercise except for daily activities	**Self-management:** After the nursing staff first instructed the patient on exercise techniques, patients can exercise on their own and can adjust the intensity and frequency of exercise according to their actual condition	HbA1c, FBG, BMI
Xueqin Lin et al.[23] (2017)/China	RCT	I:56 C:56	I:58.8 ± 5.2 C:58.5 ± 5.3	**Type:** AT **Duration:**30 min F**requency:** 3 ~ 5 times/week for 3 months	routine treatment	**Self-management:** Patients were issued "self-management cards" on which they recorded the volume and type of exercise	HbA1c, FBG, 2hPG
Suhua Yang et al.[24] (2016)/China	RCT	I:46 C:46	I:50.2 ± 9.1 C:48.9 ± 10.8	**Type:** AT **Duration:**30 ~ 60 min **Frequency:** 3 ~ 4 times/week for 12 months **Intensity:** Low/Medium intensity	routine treatment	**Family management:** The family members accompanied and supervised the patients to perform the exercise. They also recorded the time and frequency of the exercise	HbA1c, FBG, 2hPG
Haibo Wen et al.[29] (2016)/China	RCT	I:47 C:47	I:43 ~ 68 C:45 ~ 65	**Type:** AT **Duration:**40 ~ 60 min **Frequency:** ≥ 3 times/week for 6 months	Individualized pharmaceutical treatment	**Family management:** Patients appointed a family member living with them as an exercise monitor who was responsible to monitor the patient's daily exercise and record the volume of exercise and adverse reactions	HbA1c, FBG, LDL, TC, SBP, DBP
RC Plotnikoff et al.[25] (2010)/Canada	RCT	I:27 C:21	I: 55 ± 12 C: 54 ± 12	**Type:** RT **Duration:** Eight exercises were performed per session, of which, four were core exercises and four were complementary assistance exercises **Frequency:** 3 times/week for 16 weeks	Without any intervention	**Self-management:** Patients performed exercises on their own after instruction by exercise specialists and kept training logs	HbA1c, FBG, BMI, LDL, TC, HDL
J.-F.Brun et al.[26] (2008)/France	RCT	I:13 C:12	I:58.9 ± 3.6 C:60.6 ± 1.8	**Type:** AT **Duration:**45 min **Frequency:** Twice a week for 12 months	routine treatment	**Self-management:** Patients exercised at home and had to keep track of all their activity in a notebook	HbA1c, FBG, BMI, LDL, TC, HDL, SBP, DBP
Julie Ménard et al. [28](2005)/Canada	RCT	I:34 C:35	I:53.7 ± 7.5 C:55.9 ± 8.6	**Type:** CT **Duration:**45 ~ 55 min **Frequency:** 3 ~ 5 times/week for 12 months **Intensity:**50% ~ 80% HRmax	routine treatment	**Self-management:** Subjects performed the individualized home-based physical exercise program	HbA1c, FBG, BMI, LDL, TC, SBP, DBP
A.J.Van Rooijen et al. [27](2004)/South Africa	RCT	I:75 C:74	I: 40 ~ 65 C:40 ~ 65	**Type:** Walking **Duration:** 5 min/session, increasing by 10 min every two weeks, up to 45 min/day **Frequency:** ≥ 5 times/week for 12 weeks	relaxation exercise	**Self-management:** Subjects performed home-based exercises and were instructed to keep a daily record of the time they spent on each of the activities in the diary	HbA1c, BMI, SBP, DBP

*AT* Aerobic exercise intervention, *RT* Resistance exercise intervention, *CT* Combined aerobic and resistance exercise intervention, *HbA1c* Glycosylated haemoglobin, *FBG* Fasting blood glucose, *2 h PG* 2-h plasma glucose, *BMI* Body Mass Index, *SBP* Systolic blood pressure, *DBP* Diastolic blood pressure, *LDL* Low-density lipoproteins, *TG* Triglycerides, *HDL* High-density lipoproteins

### Quality Appraisal

**Fig. 2:**
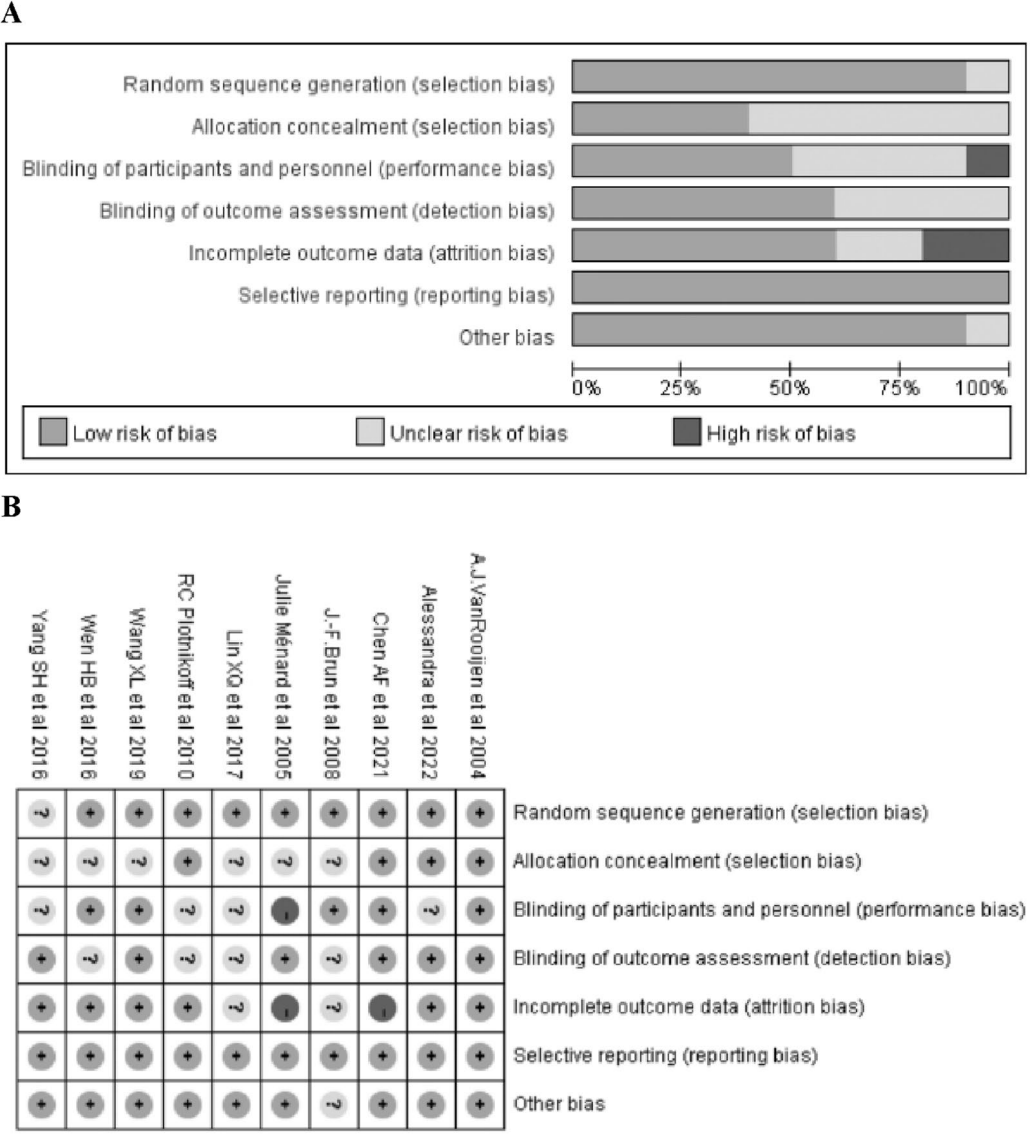
Risk of bias (ROB) assessment (**A**) ROB summary. (**B**) ROB graph

The details of the assessment are shown in Fig. 2. The method of generating random sequences was reported in 9 studies and 1 article [24] was given unclear risk because of not reporting randomization. Only 4 articles [20, 21, 25, 27] mentioned allocation concealment and none of the rest mentioned specific allocation concealment resulting in unclear risk. Only 5 articles [21, 22, 26, 27, 34] were blinded to patients and investigators, and 1 article [28] was given the high risk for not blinding, while none of the rest reported whether they were blinded to investigators and participants. Six articles [20–22, 24, 27, 28] were assessed as low risk, as objective measurement instruments were used in the outcome assessment, while the rest of the articles had subjective assessments for outcome measures, so it was unclear whether the risk of bias was present. Two articles [21, 28] were assessed as high risk because of 20% or more dropouts for attrition and missing data analysis, and 2 articles [23, 26] were assessed as the unclear risk because of lack of data on attrition rate, while the rest were assessed as low risk. All of the included articles reported prespecified outcomes and all were assessed as low risk of bias. Only 1 article[26] was assessed as unclear risk of bias for other bias, and the rest were assessed as low risk.

According to the GRADE system, low levels of certainty were shown in the results of HbA1c and FBG and very low levels of certainty were shown in the results of 2 h PG and LDL (Table 2). The downgrade was mainly due to the unclear allocation concealment in most studies, the high risk of bias in some studies, small sample sizes and wide confidence intervals, and too few studies to assess publication bias.

**Table 2:** GRADE Summary of Evidence

Certainty Assessment							No. of Patients		Effect		Certainty	Importance
No. of Studies	Study Design	Risk of Bias	Inconsistency	Indirectness	Imprecision	Other Considerations	Exercise Intervention	Usual Care	Relative (95% CI)	Absolute (95% CI)		
HbA1c 9	RCT	Serious^a,b^	Serious^c^	Not Serious	Not Serious	None	395	391	-	MD **0.81 lower** (1.21 lower to 0.40 lower)	**low**	-
FBG 9	RCT	Serious^a,b^	Serious^c^	Not Serious	Not Serious	None	388	376	-	MD **1.17 lower** (1.67 lower to 0.68 lower)	**low**	-
2h PG 3	RCT	Serious^a,b^	Serious^c^	Not Serious	Serious^d^	Publication bias strongly suspected^e^	152	152	-	MD **1.84 lower** (2.50 lower to 1.19 lower)	**Very low**	-
LDL 4	RCT	Serious^a,b^	Not Serious	Not Serious	Serious^d^	Publication bias strongly suspected^e^	121	115	-	MD **0.38 lower** (0.58 lower to 0.18 lower)	**Very low**	-

*HbA1c* glycosylated hemoglobin, *FBG* fasting blood glucose, *LDL* Low-density lipoproteins, *RCT* Randomized trials, *CI* confidence interval, *MD* mean difference

^a^ Incomplete allocation concealment

^b^ High risk of bias in one study

^c^ Substantial heterogeneity

^d^ Small sample sizes and wide confidence intervals

^e^ Too few studies to assess publication bias

### Primary outcomes (HbA1c)

#### HbA1c (%)

**Fig. 3:**
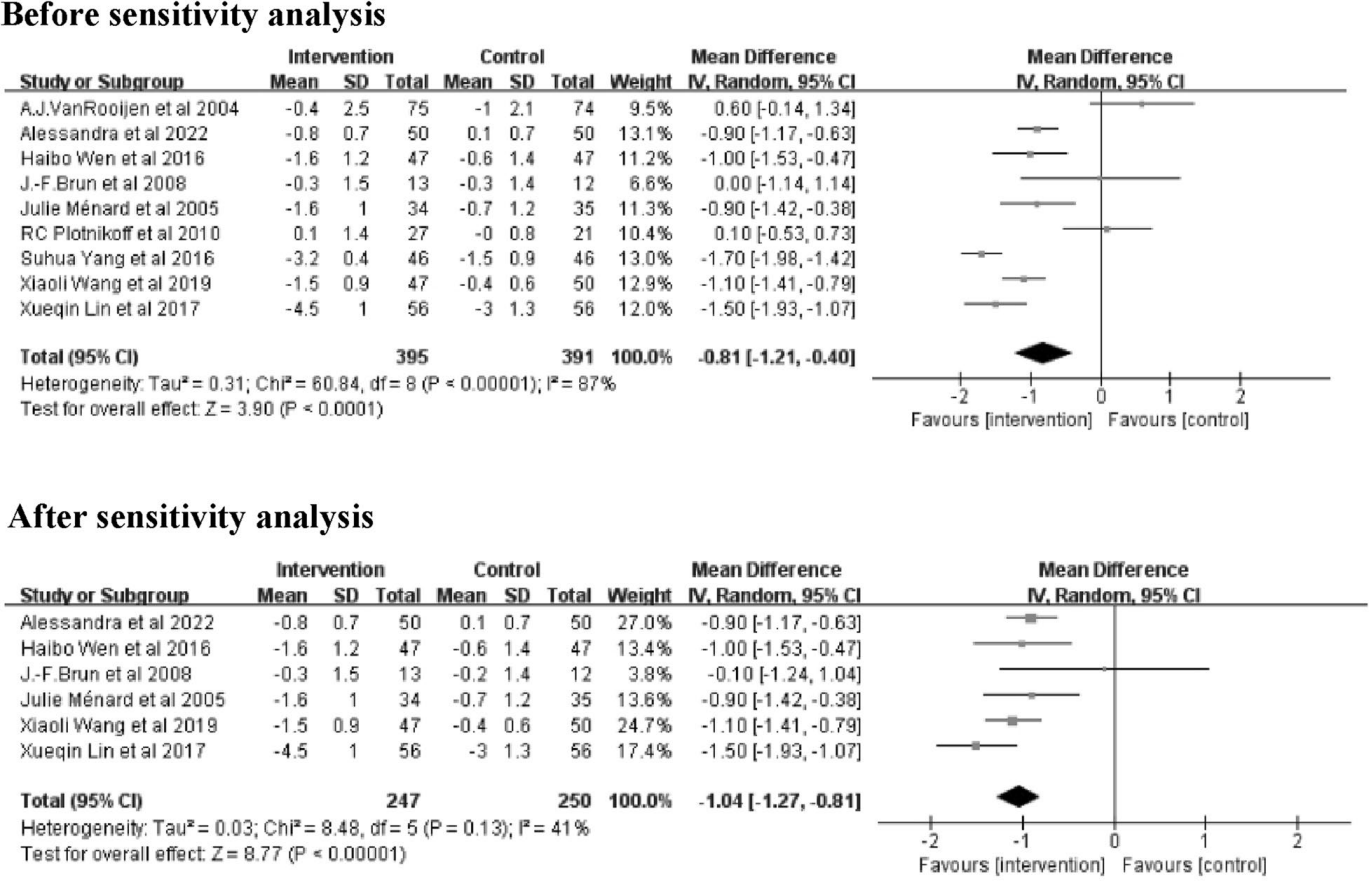
Forest plot of HbA1c on exercise interventions based on family management or self-management. HbA1c, glycosylated haemoglobin

Results from 9 studies [20, 22–29] with 786 patients were integrated to determine the effect of interventions on HbA1c levels (Fig. 3). The results indicated that, as a whole, exercise interventions based on family management or self-management significantly lowered HbA1c levels compared to the control groups (Z = 3.90; 95% *CI* *MD* = -0.81; − 1.21 to − 0.40;* P* < 0.0001). The results showed large heterogeneity (*I*^2^ = 87%). Sensitivity analysis revealed that 3 studies [24, 25, 27]were heterogeneous. After removing these 3 studies [24, 25, 27], the heterogeneity decreased to 41% with a significant effect of 1.04 (Z = 8.77; 95% *CI* − 1.27 to − 0.81; *P* < 0.00001) (Fig. 3).

### Secondary outcomes (fasting blood glucose, 2-h plasma glucose, BMI, blood pressure, lipids)

#### Fasting blood glucose (mmol/L)

**Fig. 4:**
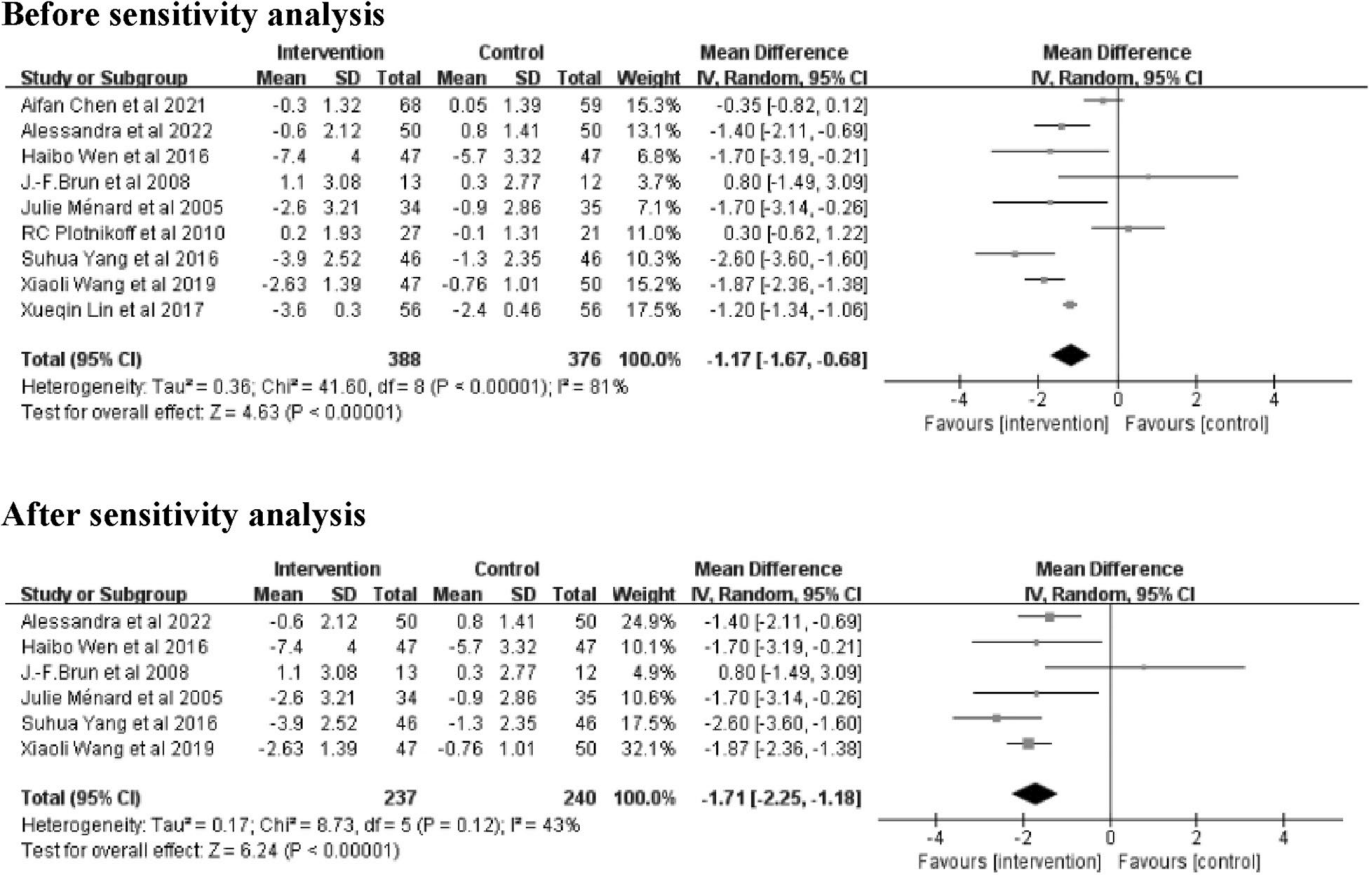
Forest plot of FBG on exercise interventions based on family management or self-management. FBG, Fasting blood glucose

The results of 9 studies [20–26, 28, 29] with 764 patients were combined to identify the effect of the Interventions on fasting blood glucose levels (Fig. 4). The results showed that, as a whole, exercise interventions based on family management or self-management significantly reduced fasting blood glucose levels compared to the control groups (Z = 4.63; 95% *CI* *MD* = − 1.17; − 1.67 to − 0.68; *P* < 0.00001). There was large heterogeneity in results (*I*^2^ = 81%). Sensitivity analysis identified 3 studies [21, 23, 25] that were heterogeneous. After removing these 3 studies [21, 23, 25], the heterogeneity decreased to 43% with a significant effect of 1.71(Z = 6.24; 95% *CI* − 2.25 to − 1.18; *P* < 0.00001) (Fig. 4).

#### 2-h plasma glucose (mmol/L)

**Fig. 5:**
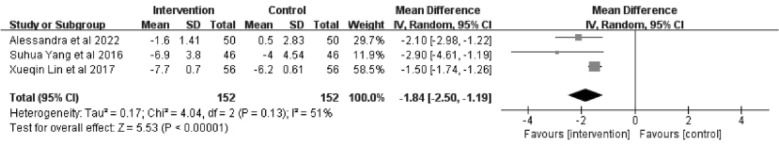
Forest plot of 2 h PG on exercise interventions based on family management or self-management. 2 h PG, 2-h plasma glucose

Three studies [20, 23, 24] reporting 2-h plasma glucose levels had a total of 304 patients included in the studies. The results revealed a significant difference between the intervention and control groups (Z = 5.53; 95% *CI*; *MD* = − 1.84; − 2.50 to − 1.19; *P* < 0.00001). There was heterogeneity between studies (*I*^2^ = 51%) (Fig. 5).

#### Body mass index (BMI) (kg/m.^2^)

**Fig. 6:**
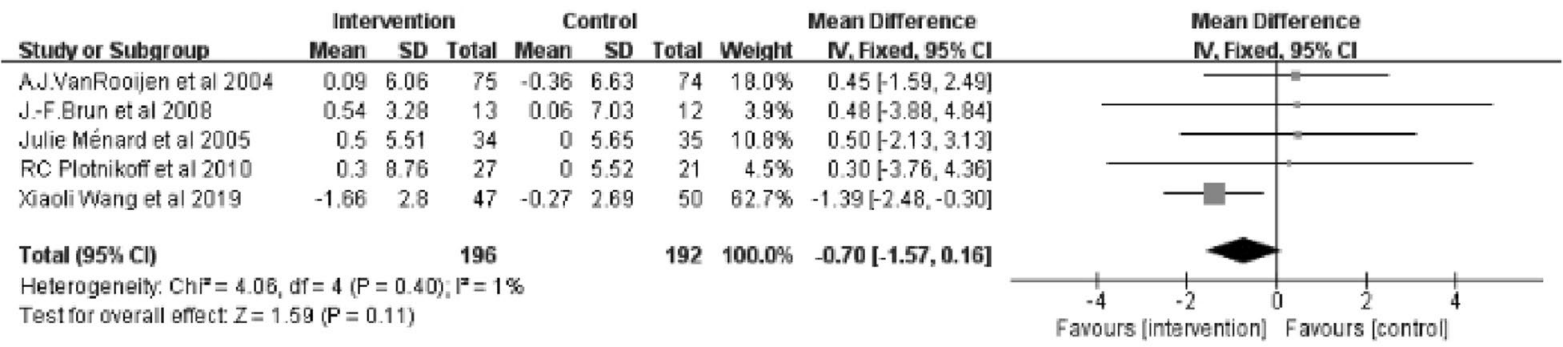
Forest plot of BMI on exercise interventions based on family management or self-management

Five studies [22, 25–28] reporting on BMI with a total of 388 patients were included in the studies. The results of the Meta-analysis showed no significant differences between the intervention and control groups (Z = 1.59; 95% *CI* *MD* = − 0.70; − 1.57 to 0.16; P = 0.11). And there was almost no heterogeneity between studies (*I*^2^ = 1%) (Fig. 6).

#### Blood pressure (BP) (mmHg)

**Fig. 7:**
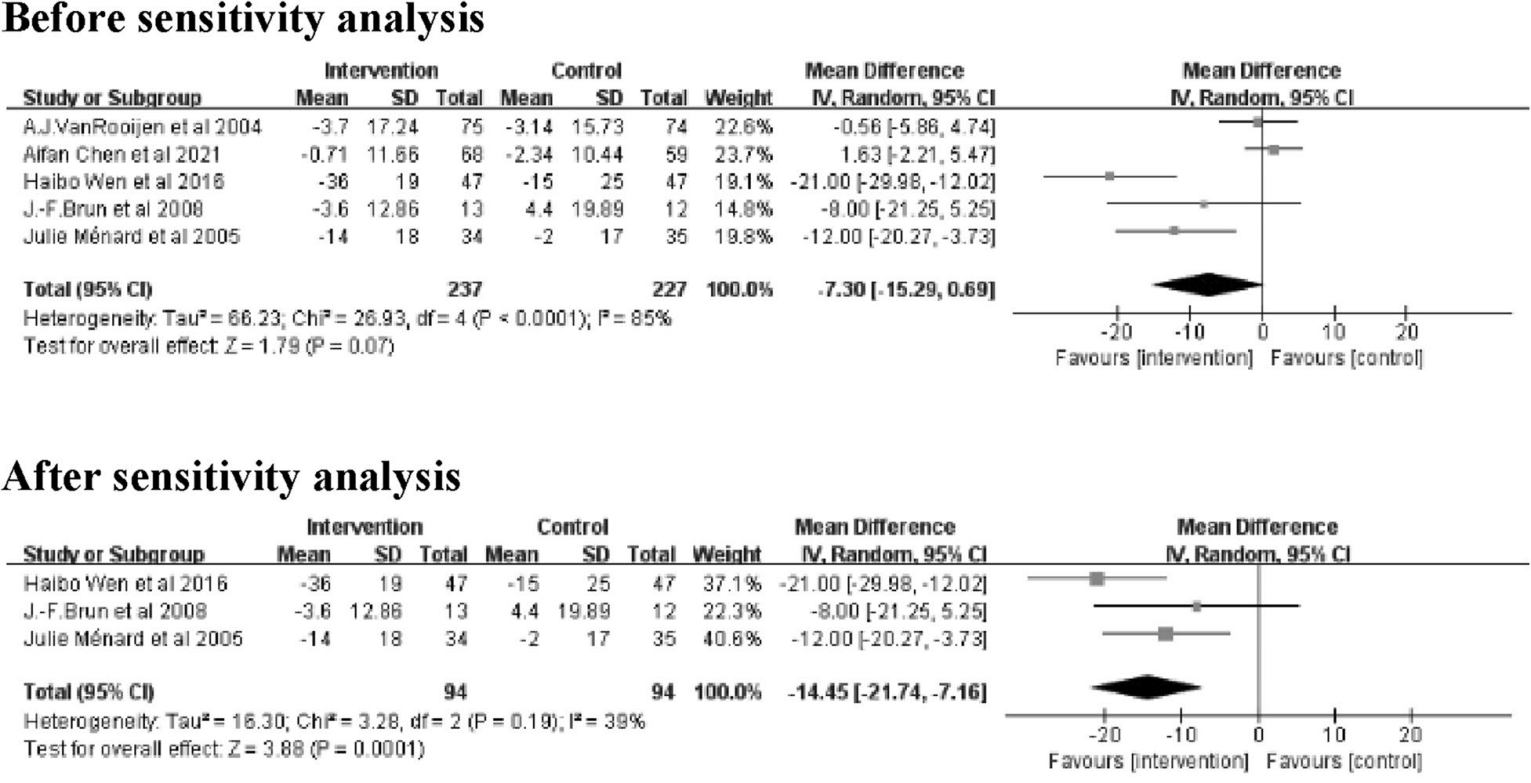
Forest plot of SBP on exercise interventions based on family management or self-management. SBP, Systolic blood pressure

The meta-analysis for systolic blood pressure (SBP) and diastolic blood pressure (DBP) included 5 studies [21, 26–29] with 464 patients in total. Meta-analysis of SBP showed no significant differences between the intervention and control groups (Z = 1.79; 95% *CI* *MD* = − 7.30; − 15.29 to 0.69; *P* = 0.07). The results showed large heterogeneity (I^2^ = 85%). Sensitivity analysis revealed that the 2 studies [21, 27] were heterogeneous. After removing these 2 studies [21, 27], the heterogeneity decreased to 39% and there was a significant difference between the intervention and control groups (Z = 3.88; 95% *CI* *MD* = − 14.45; − 21.74 to − 7.16; *P* = 0.0001) (Fig. 7).

**Fig. 8:**
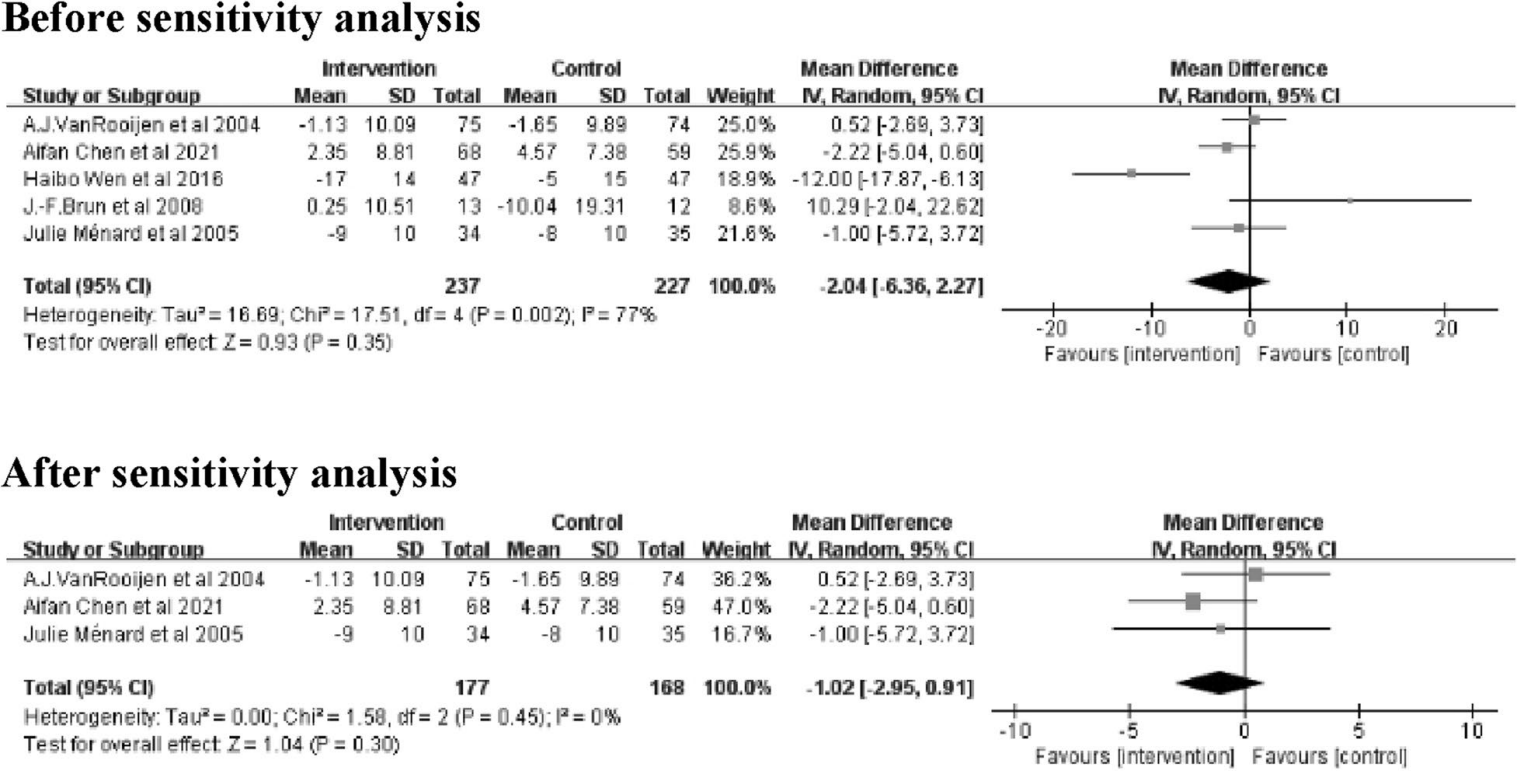
Forest plot of DBP on exercise interventions based on family management or self-management. DBP, Diastolic blood pressure

Meta-analysis of DBP showed no significant differences between the intervention and control groups (Z = 0.93; 95% *CI* *MD* = − 2.04; − 6.36 to 2.27;* P* = 0.35). The results showed large heterogeneity (*I*^2^ = 77%). Sensitivity analysis revealed that the 2 studies [26, 29] were heterogeneous. After removing these 2 studies [26, 29], heterogeneity was completely reduced (I^2^ = 0%), but there was still no significant difference between the intervention and control groups (Z = 1.04; 95% *CI*; *MD* = -1.02; -2.95 to 0.91; *P* = 0.30) (Fig. 8).

#### Lipids (mmol/L)

**Fig. 9:**
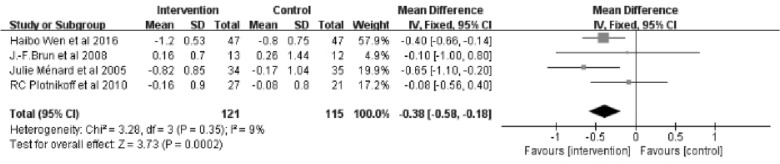
Forest plot of LDL on exercise interventions based on family management or self-management. LDL, Low-density lipoproteins

The meta-analysis for Low-density lipoproteins consisted of 4 studies [25, 26, 28, 29] with a total of 236 patients. The results indicated a significant difference between the intervention and control groups (Z = 3.73; 95% *CI* *MD* = − 0.38; − 0.58 to − 0.18; *P* = 0.0002). The results showed no significant heterogeneity between studies (*I*^2^ = 9%) (Fig. 9).

**Fig. 10:**
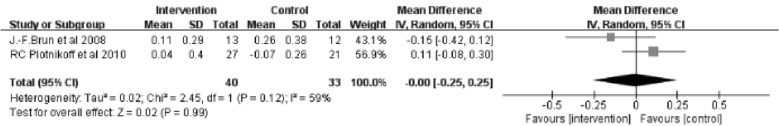
Forest plot of HDL on exercise interventions based on family management or self-management. HDL, High-density lipoproteins

The meta-analysis for High-density lipoproteins consisted of 2 studies [25, 26] with a total of 73 patients. The Meta-analysis of High-density lipoproteins showed no significant differences between the intervention and control groups (Z = 0.02; 95% *CI* *MD* = 0.00; − 0.25 to 0.25; *P* = 0.99). There was heterogeneity between studies (*I*^2^ = 59%) (Fig. 10).

**Fig. 11:**
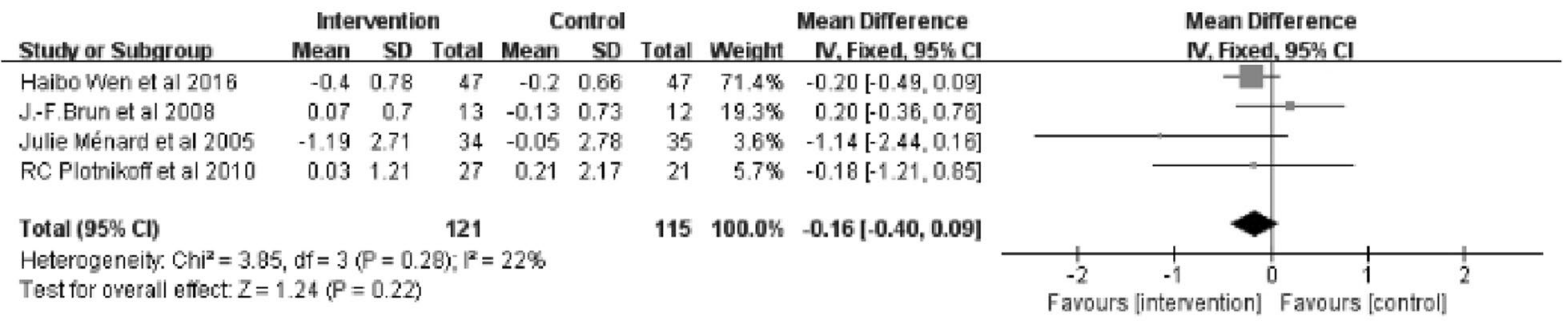
Forest plot of TG on exercise interventions based on family management or self-management. TG, Triglycerides

The meta-analysis for triglycerides included 4 studies [25, 26, 28, 29] with a total of 236 patients. The Meta-analysis of triglycerides showed no significant differences between the intervention and control groups (Z = 1.24; 95% *CI* *MD* = − 0.16; − 0.40 to 0.09; *P* = 0.22). The results showed no significant heterogeneity between studies (*I*^2^ = 22%) (Fig. 11).

#### Subgroup analysis

Subgroup analysis were conducted according to exercise frequency (3 times/week; > 3 times/week), types of exercise (aerobic exercise; aerobic combined resistance exercise), duration of intervention (short- to medium-term: 3–6 months; long-term: 12 months), and baseline levels of HbA1c (≤ 7.5%, > 7.5%) and fasting blood glucose (≤ 7.5 mmol/L, > 7.5 mmol/L). Due to the insufficient number of studies included in each subgroup for the other outcomes, subgroup analyses were only conducted for fasting blood glucose and HbA1c according to the frequency of exercise and types of exercise. The subgroup analyses based on exercise frequency found that the exercise frequency had no bearing on the effect sizes for fasting blood glucose (subgroup difference *P* = 0.16) and HbA1c (subgroup difference *P* = 0.28). Both three and more than three times per week of exercise based on family or self-management improved HbA1c levels in patients with T2DM. For fasting blood glucose, we only found that performing home-based or self-managed exercise more than three times per week caused a reduction in fasting blood glucose. However, the effect of exercise performed three (*P* = 0.17) times per week on fasting blood glucose was not significant (Table 3).

**Table 3:** Summary of subgroup analysis results

Outcome	Number of RCTs	Number of participants	Heterogeneity		Meta analysis		subgroup differences
			*P* value	*I*^*2*^ (%)	Effect estimate (95% *CI*)	*P* value	
The meta-analysis results by exercise frequency							
HbA1c (%)	8[20, 22–25, 27–29]	761	< 0.00001	88	− 0.87[− 1.28, − 0.45]	< 0.0001	0.39
3 times/week	3[20, 25, 29]	242	0.01	78	− 0.65[− 1.22, − 0.08]	0.03	
> 3 times/week	5[22–24, 27, 28]	519	< 0.00001	89	− 0.99[− 1.55, − 0.43]	0.0005	
FBG (mmol/L)	8[20–25, 28, 29]	739	< 0.00001	82	− 1.25[− 1.75, − 0.75]	< 0.00001	0.44
3 times/week	3[20, 25, 29]	242	< 0.0001	79	− 0.88[− 2.14,0.37]	0.17	
> 3 times/week	5[21–24, 28]	497	< 0.00001	86	− 1.43[− 2.03,− 0.83]	< 0.00001	
The meta-analysis results by types of exercise							
HbA1c (%)	8[20, 22–24, 26–29]	738	< 0.00001	85	− 0.93[− 1.32,− 0.54]	< 0.00001	1.00
AT	6[22–24, 26, 27, 29]	569	< 0.00001	88	− 0.90[− 1.44,− 0.36]	0.001	
CT	2[20, 28]	169	1.00	0	− 0.90[− 1.14,− 0.66]	< 0.00001	
FBG (mmol/L)	8[20–24, 26, 28, 29]	716	< 0.0001	78	− 1.35[− 1.83,− 0.87]	< 0.00001	0.74
AT	6[21–24, 26, 29]	547	< 0.0001	84	− 1.31[− 1.91,− 0.71]	< 0.0001	
CT	2[20, 28]	169	0.71	0	− 1.46[− 2.09,− 0.82]	< 0.00001	
The meta-analysis results by the durations of exercise interventions							
HbA1c (%)	9[20, 22–29]	786	< 0.00001	87	− 0.81[− 1.21,− 0.40]	< 0.00001	0.26
Short-to-medium term	4[23, 25, 27, 29]	403	0.0002	91	− 0.48[− 1.41,0.44]	0.31	
Long term	5[20, 22, 24, 26, 28]	383	< 0.00001	82	− 1.06[− 1.47,− 0.65]	< 0.0001	
FBG (mmol/L)	9[20–26, 28, 29]	764	< 0.00001	81	− 1.17[− 1.67,− 0.68]	< 0.00001	0.45
Short-to-medium term	3[23, 25, 29]	254	0.005	81	− 0.82[− 1.90,0.25]	0.13	
Long term	6[20–22, 24, 26, 28]	510	< 0.0001	84	− 1.34[− 2.16,− 0.53]	0.001	
LDL (mmol/L)	4[25, 26, 28, 29]	236	0.35	9	− 0.38[− 0.58,− 0.18]	0.0002	0.36
Short-to-medium term	2[25, 29]	142	0.25	23	− 0.33[− 0.56,− 0.10]	0.005	
Long term	2[26, 28]	94	0.28	13	− 0.54[− 0.94,− 0.14]	0.008	
TG (mmol/L)	4[25, 26, 28, 29]	236	0.28	22	− 0.16[− 0.40,0.09]	0.17	0.53
Short-to-medium term	2[25, 29]	142	0.97	0	− 0.20[− 0.48,0.08]	0.96	
Long term	2[26, 28]	94	0.06	71	− 0.01[− 0.53,0.50]	0.22	
SBP (mmHg)	5[21, 26–29]	464	< 0.0001	85	− 7.30[− 15.29,0.69]	0.07	0.66
Short-to-medium term	2[27, 29]	243	0.0001	93	− 10.45[− 30.47,9.57]	0.31	
Long term	3[21, 26, 28]	221	0.008	79	− 5.38[− 15.44,4.68]	0.29	
DBP (mmHg)	5[21, 26–29]	464	0.002	77	− 2.04[− 6.36,2.27]	0.38	0.46
Short-to-medium term	2[27, 29]	243	0.0002	93	− 5.49[− 17.75,6.77]	0.79	
Long term	3[21, 26, 28]	221	0.15	47	− 0.57[− 4.68,3.54]	0.35	
BMI (kg/m^2^)	5[22, 25–28]	388	0.40	1	-0.70[-1.57,0.16]	0.11	0.17
Short-to-medium term	2[25, 27]	197	0.95	0	0.42[-1.40,2.24]	0.65	
Long term	3[22, 26, 28]	191	0.34	8	− 1.03[− 2.01,− 0.05]	0.04	
The meta-analysis results by baseline levels							
HbA1c (%)	9[20, 22–29]	786	< 0.00001	87	− 0.81[− 1.21,− 0.40]	< 0.0001	0.99
≤ 7.5%	4[20, 22, 25, 29]	339	0.009	74	− 0.79[− 1.19,− 0.40]	< 0.0001	
> 7.5%	5[23, 24, 26–28]	447	< 0.00001	90	− 0.79[− 1.53,− 0.05]	0.04	
FBG (mmol/L)	9[20–26, 28, 29]	764	< 0.00001	81		< 0.00001	0.04
≤ 7.5mmol/L	3[20, 21, 25]	275	0.009	79		0.24	
> 7.5mmol/L	6[22–24, 26, 28, 29]	489	0.04	71		< 0.00001	

*HbA1c* Glycosylated haemoglobin, *FBG* Fasting blood glucose, *AT* Aerobic exercise intervention, *RT* Resistance exercise intervention, *CT* Combined aerobic and resistance exercise intervention, *BMI* Body Mass Index, *SBP* Systolic blood pressure, *DBP* Diastolic blood pressure, *LDL* Low-density lipoproteins, *TG* Triglycerides

Subgroup analysis based on exercise types revealed that effect sizes for HbA1c (subgroup difference P = 1.00) and fasting glucose (subgroup difference P = 0.7) were not affected by exercise types. Both aerobic exercise interventions and aerobic combined with resistance exercise interventions improved HbA1c and fasting blood glucose levels in T2DM following family or self-management (Table 3).

Since the number of studies included in each subgroup for 2-h plasma glucose and HDL was too limited, subgroup analyses were conducted for the other seven outcomes according to the duration of the intervention. The subgroup analysis revealed that the duration of the intervention had no bearing on the effect sizes for any of the outcomes. Additionally, we discovered that the 12-month intervention duration significantly lowered levels of HbA1c (*P* < 0.0001), fasting blood glucose (*P* = 0.001), and BMI (*P* = 0.04). However, the impact of 3–6 months of intervention duration on HbA1c (*P* = 0.31), fasting blood glucose (*P* = 0.13) and BMI (*P* = 0.65) levels was not significant (Table 3).

Subgroup analysis based on baseline levels found that the effect size for HbA1c (subgroup difference P = 0.99) was not impacted by baseline levels. The improvements in fasting blood glucose were more significant with the exercise interventions based on family or self-management when the baseline level of fasting blood glucose in T2DM was > 7.5 mmol/L (*P* < 0.00001). However, this study did not find a significant reduction in T2DM with a baseline level of fasting glucose ≤ 7.5 mmol/L by exercise interventions based on family or self-management (*P* = 0.24) (Table 3).

### Sensitivity analysis

Sensitivity analyses were performed by excluding the studies one by one. After removing studies with heterogeneity, except for systolic blood pressure, the intervention results for all outcomes remained largely consistent with those when not previously excluded, which suggested that the results were stable and reliable. In addition, this study assessed the effect of single exercise session duration based on family or self-management on HbA1c and FBG by sensitivity analysis. The article by RC Plotnikoff et al. [25] did not state the specific duration of the exercise, whereas the single exercise session of the other articles was 30–60 min. Therefore, this article [25] was excluded from the sensitivity analysis. The findings revealed that exercise for 30–60 min at a time, with family or self-management, significantly improved HbA1c (Z = 4.65; 95% CI MD = − 0.93; − 1.32 to − 0.54; *P* < 0.00001) and FBG (Z = 5.49; 95% CI MD = − 1.35;− 1.83 to -0.87; *P* < 0.00001) levels.

### Adverse events

In the 10 included studies, the occurrence of adverse events was explicitly reported in 2 studies [28, 29]. One study [28] reported that at least one minor hypoglycemic episode was experienced by 42% of patients per month. The intervention group reported 3 severe hypoglycemic episodes (1 concurrent with acute alcohol intoxication). Each group reported 2 non-fatal cardiac events. Another study[29] reported 3 cases of hypoglycemia and 1 case each of angina pectoris and hypertensive crisis in the intervention group and 1 case each of hypoglycemia and hypertensive crisis in the control group. However, none of them occurred again after treatment and adjustment of the exercise volume. No adverse events were reported in any of the other eight studies.

### Publication bias

Egger's asymmetry test and funnel plots were conducted in outcomes with data from close to 10 studies(HbA1c, Fasting blood glucose) [33]. Egger’s asymmetry tests and funnel plots revealed little indication of publication bias for HbA1c and fasting blood glucose *(P* = 0.053 for HbA1c; *P* = 0.906 for fasting blood glucose). The results of HbA1c were adjusted by the trim and fill method. It was found that the number of studies and results were unchanged, suggesting stable and reliable results for HbA1c.

## Discussion

Exercise intervention is a generic term, it refers to all physical activities that increase energy expenditure and is an important component in the control and management of T2DM[35]. Many meta-analyses and systematic reviews [5, 6, 14] have demonstrated that exercise not only has significant benefits on glucolipid metabolism and other health outcomes in patients with T2DM, but also effectively prevents and delays the complications of T2DM and improves the quality of life of patients with T2DM. Therefore, if people with T2DM can make exercise a part of their lives and keep it for a long time, they could effectively improve their condition.

Results from a Meta-analysis of 10 randomized controlled trials including 913 participants showed that, overall, compared to control groups, exercise interventions based on family management or self-management significantly reduced levels of HbA1c, fasting blood glucose, 2-h plasma glucose, and Low-density lipoproteins in T2DM patients [20–29]. The results of our meta-analysis are broadly consistent with some systematic reviews and meta-analysis reports on the effects of exercise on glycaemic responses in patients with T2DM [36, 37]. A systematic review [14] has shown that autonomous exercise is beneficial for the improvement of HbA1c and other biomarkers in patients with T2DM. Most of the T2DM patients in the included RCTs had comorbidities such as obesity, hypertension, and dyslipidaemia. Therefore, the meta-analysis was performed with lipids, blood pressure and BMI as secondary outcomes. Regarding the results of the meta-analysis on lipids, we only found that exercise interventions based on family or self-management had a significant effect on low-density lipoprotein levels in T2DM, while the effects on other lipid markers were not significant. This was consistent with the results of a previous meta-analysis[38]. The results for blood pressure and BMI were different from those of previous studies. In this review, we did not find a significant effect on BMI and blood pressure in T2DM from exercise interventions based on family or self-management. However, some meta-analyses [36, 39, 40] showed that exercise had a significant effect on lowering blood pressure and BMI. The reason for the different results may be partly due to the small number of included studies and partly due to the low baseline levels of blood pressure in the included studies, resulting in a non-significant difference before and after the intervention.

We found that exercise interventions based on family or self-management reduced HbA1c by 0.81%. The UK Prospective Diabetes Study (UKPDS) in Type 2 diabetes [41] has documented that every 1% reduction in mean HbA1c levels decreased the risk of diabetes-related death by 21%, the risk of myocardial infarction by 14% and microvascular complications by 37%. Applying this data to the findings of the current study, exercise interventions based on family or self-management can reduce the risk of death due to diabetes by 17%, reduce the risk of myocardial infarction by 11% and microvascular complications by 30%. Additionally, we discovered that the effect size of HbA1c was not affected by the frequency of exercise and that it required a longer exercise intervention (> 6 months) to improve HbA1c.

Exercise interventions based on family or self-management decreased fasting blood glucose by 1.17 mmol/L and 2-h plasma glucose by 1.84 mmol/L. According to the DECODE Mortality Follow-up Study [42], sudden death could be prevented by a 2-mmol/L reduction in 2-h plasma glucose. In the present study, the 2-h plasma glucose in the intervention groups decreased by 1.84 mmol/L after the interventions, which was close to 2 mmol/L. This study [42] also revealed an increase in all-cause mortality following fasting blood glucose rises above 7.0 mmol/L and a linear increase in mortality following 2-h plasma glucose elevations above 4.5 mmol/L. In the intervention groups of the included studies, patients with type 2 diabetes had a mean baseline fasting blood glucose level of 9.6 mmol/L and a mean baseline 2-h plasma glucose level of 15.9 mmol/L. Therefore, in the present study, improving fasting blood glucose and 2-h plasma glucose levels in patients with type 2 diabetes reduced all-cause mortality. We also found that it took more than three exercise sessions per week and a longer duration of exercise intervention (> 6 months) to lower fasting blood glucose.

Exercise interventions based on family or self-management lowered LDL by 0.38 mmol/L. According to a recent meta-analysis [43], coronary heart disease deaths decreased by 22%, major cardiovascular events decreased by 21%, and all-cause mortality decreased by 9% with every 1 mmol/L reduction in LDL. Regarding exercise interventions based on family or self-management, this would translate into an 8.4% reduction in coronary heart disease mortality, an 8.0% reduction in major cardiovascular event rates, and a 3.4% reduction in all-cause mortality. Compared to statins (− 0.19 mmol/L), fibrates (− 0.23 mmol/L), and diet/surgery (− 0.17 mmol/L) [44], this has a greater impact on LDL. We also found that the effect size of LDL was not affected by the duration of the intervention.

The meta-analysis of some outcomes also displayed high heterogeneity. Therefore, sensitivity analyses were conducted to find the sources of heterogeneity. Sensitivity analysis of HbA1c revealed that the studies of RC Plotnikoff et al. [25], A.J. Van Rooijen et al. [27], and Suhua Yang et al. [24] were the main sources of heterogeneity. In the study by RC Plotnikoff et al. [25], baseline levels of HbA1c were relatively low in patients with T2DM, with a mean HbA1c of only 6.89 ± 1.5% in the resistance exercise group. The other potential reason may be connected to the non-significant increase in lean body mass with resistance exercise in this study. Previous studies [45, 46] have suggested a substantial negative association between increased muscle mass through resistance exercise and the decrease in HbA1c and fasting glucose. In the study by A.J. Van Rooijen et al. [27], participants did too little exercise per week, averaging a total of about 30 min of moderate-intensity aerobic training per week. Whereas HbA1c reflects the average blood glucose levels of the body over the past three months, too little exercise may not be enough to cause a relatively considerable improvement in the HbA1c of the body. In the study by Suhua Yang et al. [24], baseline levels of HbA1c were too high relative to several other studies, which may have led to more significant improvements in HbA1c. In addition, this study [24] did not indicate whether participants and investigators were blinded, which may have resulted in a more significant change in HbA1c due to the investigators' interest in the study and the subjective positive effect of the participants. Therefore, these three studies were excluded during sensitivity analysis.

Sensitivity analysis of fasting blood glucose revealed high heterogeneity resulting from the studies of RC Plotnikoff et al. [25], Aifan Chen et al. [21], and Xueqin Lin et al. [23]. In the study of RC Plotnikoff et al. [25], the baseline level of fasting blood glucose in the resistance exercise group was already inherently lower than in the other studies, at 6.9 ± 2.1 mmol/L, which may have led to an insignificant reduction in fasting blood glucose. The other potential reason may also be connected to the non-significant increase in lean body mass with resistance exercise in this study. The reason for the heterogeneity in the study by Xueqin Lin et al. [23] maybe related to the duration of the intervention. The duration of the intervention in this study was 3 months, while most of the other included studies had intervention duration of 12 months. For this reason, although the study reduced fasting blood glucose, the effect was not significant compared to other studies, which caused heterogeneity among the studies. In the study by Aifan Chen et al. [21], the baseline level of fasting glucose in the intervention group was lower, with mean fasting glucose of 7.21 mmol/L, which may have made the difference before and after the intervention insignificant, thus showing considerable heterogeneity. Therefore, these three studies were excluded during sensitivity analysis.

The sensitivity analysis of SBP revealed high heterogeneity in the studies by Aifan Chen et al. [21] and A.J. Van Rooijen et al. [27]. The main reason for the heterogeneity may be due to the lower baseline SBP levels in the intervention groups in these two studies compared to the other studies included. The SBP was only 137.48 ± 12.96 mmHg in the study by Aifan Chen et al. [21] and 131.81 ± 18.07 mmHg in the study by A.J. Van Rooijen et al. [27]. Therefore, these two studies were excluded during sensitivity analysis. And the results of the meta-analysis of SBP showed that the SBP in the intervention group was lower than that in the control group (P = 0.0001). In contrast, the reduction in SBP was not significant before the sensitivity analysis (P = 0.07). Therefore, the results of SBP need to be explained with caution.

The sensitivity analysis of DBP revealed that the studies of J.-F. Brun et al. [26] and Haibo Wen et al. [29]were the main sources of heterogeneity. The reason for the heterogeneity in the study by J.-F. Brun et al. [26] is the baseline level of DBP in that study was relatively low, with a DBP of only 78.21 ± 10.3 mm Hg in the intervention group. The other reason is the protocol design of that study by J.-F. Brun et al. did not control for blood pressure, the intervention may have been reduced or even suspended when there was a decrease in blood pressure [26].In the study by Haibo Wen et al. [29], patients with T2DM combined with hypertension were included, so the baseline level of DBP in that study would have been much higher than in the other studies. The results of this study [29] also showed a more significant decrease in DBP compared to other included studies, which led to heterogeneity with other studies. Therefore, these two studies were excluded during sensitivity analysis.

This review is the first meta-analysis to systematically evaluate the effect of exercise interventions based on family management or self-management on glycaemic control in patients with T2DM. Most of the RCTs included in previous Meta-analyses have performed exercise interventions in laboratories or hospitals [5, 6, 40]. Hospitals or laboratories tend to have specialised instrumentation and patients with T2DM are supervised by professionals. Exercise interventions conducted in such environments may result in better outcomes and adherence for patients. It is uncertain whether the results of such findings can be applied on a large scale to the real-life situation of T2DM. The present review explored the effects of exercise interventions based on family or self-management on T2DM in a community setting. According to the studies included in this review, the specific management style of family or self-management mainly involved the patients with T2DM themselves or accompanied by family members performed the exercise intervention after the exercise intervention programme was developed by professionals. The family members or the patients themselves were responsible for recording and monitoring the exercise. It was this exercise intervention management in the living settings in which people with T2DM are living that would be more conducive to our understanding of the feasibility of this model of exercise intervention management in the real-world context.

### Study limitations

There are some limitations to this study. First, as shown in previous reports, the quality of the included studies was mostly evaluated as moderate risk of bias. Many of the studies were not described in terms of allocation concealment, study personnel, and subject administration blinding, so it was unclear whether there was a risk of bias, which also led to a lack of rigour in the description and design of some trials. Second, the number of included RCTs in this study was infrequent and only articles in English and Chinese were selected, which may limit the suitability of the study results. Last, some of the included studies did not describe exercise intensity, and others described exercise intensity in a way that was difficult to standardise. This study was unable to investigate the effects of exercise intensity on this model of exercise intervention management. Therefore, more high-quality studies evaluating exercise interventions based on family management or self- management are needed, which should be important for the development of clinical guidelines for the care and management of T2DM.

### Practical implications

Nowadays, medical resources were limited and medical staff was in heavy demand [47]. One of the significant advantages of exercise interventions based on family or self-management is that they can reduce the need for medical staff to supervise patients withT2DM, thereby reducing the burden of medical treatment and improving condition management's cost-effectiveness and practicability [48, 49].Based on the evidence, we recommended that the exercise prescription can be set according to more than three times of exercise per week for 30–60 min per session in exercise interventions based on family or self-management for T2DM in the future, which is analogous to the exercise guidelines issued by the American Diabetes Association (ADA) [7].This also demonstrates that exercise interventions based on family or self-management need to be built up over time and cycles before better results can occur. As for the types of exercise, this review recommended that patients with T2DM could do aerobic exercise interventions or aerobic combined with resistance exercise interventions, with family or self-management.

In addition, we recommend more than 6 months exercise interventions if the patients are exercising under family or self-management. This review did not discover that 3–6 months of exercise intervention led to improvements in HbA1c, FBG and BMI levels. This was different from the results of the previous meta-analysis [50]. It may be related to the characteristics of family or self-management. Patients with T2DM and family members may lack relevant expertise, resulting in less efficient management and requiring longer management of exercise interventions to elicit a clinical reduction in blood glucose. In order to shorten the intervention duration, perhaps the exercise intervention management for T2DM could be in the form of mainly family or self-management, supplemented by the guidance of community physicians. During family or self-management, the community physicians will follow up with the patients and their families on a regular basis to provide exercise guidance and to address any problems that may exist, thus enhancing the efficiency of family or self-management.

## Conclusions

Exercise interventions based on family management or self-management can significantly reduce HbA1c, fasting glucose, 2-h plasma glucose, and Low-density lipoproteins levels in patients with T2DM, which can effectively delay disease progression and reduce the risk of developing complications. T2DM patients themselves or their family members should perform exercise interventions according to the exercise intervention programmes made by professionals, and supervise and manage every exercise intervention by recording it. In the future, for exercise interventions based on family or self-management, this review recommended that exercise intervention programmes should be formulated according to 30–60 min per session, more than three times per week, for more than six months of aerobic exercise or aerobic combined with resistance exercise.

### Supplementary Information

**Additional file 1: **Full Search Queries Used in Pubmed, Web of Science, The Cochrane Library, and Embase (Updated October 17, 2022).

**Additional file 2: **PRISMA 2020 checklist

**Additional file 3: **Baseline data for all outcomes

## Data Availability

All data generated or analysed during this study are included in this published article (and its Additional files).

## Abbreviations

T2DM: Type 2 diabetes mellitus
HbA1c: Glycosylated haemoglobin
FBG: Fasting blood glucose
2hPG: 2-H plasma glucose
BMI: Body Mass Index
SBP: Systolic blood pressure
DBP: Diastolic blood pressure
LDL: Low-density lipoproteins
TG: Triglycerides
HDL: High-density lipoproteins
AT: Aerobic exercise intervention
RT: Resistance exercise intervention
CT: Combined aerobic and resistance exercise intervention
