# Supplementary material for: Effect of exercise interventions based on family management or self-management on glycaemic control in patients with type 2 diabetes mellitus: a systematic review and meta-analysis
Source: Diabetol Metab Syndr. 2023 Nov 14;15:232. doi: 10.1186/s13098-023-01209-4 (PMC10644553; doi:10.1186/s13098-023-01209-4)
Supplement: Supplementary file 1 — Additional file 1: Full Search Queries Used in Pubmed, Web of Science, The Cochrane Library, and Embase (Updated October 17, 2022). [file 13098_2023_1209_MOESM1_ESM.docx]

**Additional file 1**

**Additional Table 1**

Full Search Queries Used in Pubmed (Updated October 17, 2022)

| Search | Query | Results |
| --- | --- | --- |
| #1 | "Diabetes Mellitus, Type 2"[Mesh] | 162229 |
| #2 | Diabetes Mellitus, Noninsulin-Dependent[Title/Abstract] OR Diabetes Mellitus, Ketosis-Resistant[Title/Abstract] OR Diabetes Mellitus, Ketosis Resistant[Title/Abstract] OR Ketosis-Resistant Diabetes Mellitus[Title/Abstract] OR Diabetes Mellitus, Non Insulin Dependent[Title/Abstract] OR Diabetes Mellitus, Non-Insulin-Dependent[Title/Abstract] OR Non-Insulin-Dependent Diabetes Mellitus[Title/Abstract] OR Diabetes Mellitus, Stable[Title/Abstract] OR Stable Diabetes Mellitus[Title/Abstract] OR Diabetes Mellitus, Type II[Title/Abstract] OR NIDDM[Title/Abstract] OR Diabetes Mellitus, Noninsulin Dependent[Title/Abstract] OR Diabetes Mellitus, Maturity-Onset[Title/Abstract] OR Diabetes Mellitus, Maturity Onset[Title/Abstract] OR Maturity-Onset Diabetes Mellitus[Title/Abstract] OR Maturity Onset Diabetes Mellitus[Title/Abstract] OR Type 2 Diabetes Mellitus[Title/Abstract] OR Noninsulin-Dependent Diabetes Mellitus[Title/Abstract] OR Noninsulin Dependent Diabetes Mellitus[Title/Abstract] OR Maturity-Onset Diabetes[Title/Abstract] OR Diabetes, Maturity-Onset[Title/Abstract] OR Maturity Onset Diabetes[Title/Abstract] OR Type 2 Diabetes[Title/Abstract] OR Diabetes, Type 2[Title/Abstract] OR Diabetes Mellitus, Adult-Onset[Title/Abstract] OR Adult-Onset Diabetes Mellitus[Title/Abstract] OR Diabetes Mellitus, Adult Onset[Title/Abstract] | 169737 |
| #3 | #1 OR #2 | 220713 |
| #4 | "Exercise"[Mesh] | 236671 |
| #5 | Exercises[Title/Abstract] OR Physical Activity[Title/Abstract] OR Activities, Physical[Title/Abstract] OR Activity, Physical[Title/Abstract] OR Physical Activities[Title/Abstract] OR Exercise, Physical[Title/Abstract] OR Exercises, Physical[Title/Abstract] OR Physical Exercise[Title/Abstract] OR Physical Exercises[Title/Abstract] OR Exercise, Acute[Title/Abstract] OR Exercises, Acute[Title/Abstract] OR Exercise Training[Title/Abstract] OR Exercise Trainings[Title/Abstract] OR Training, Exercise[Title/Abstract] OR Trainings, Exercise OR[Title/Abstract] | 272751 |
| #6 | #4 OR #5 | 403269 |
| #7 | "Community Health Planning"[Mesh] | 5235 |
| #8 | Community Health Plannings[Title/Abstract] OR Health Planning, Community[Title/Abstract] OR Health Plannings, Community[Title/Abstract] OR Planning, Community Health[Title/Abstract] OR Plannings, Community Health[Title/Abstract] OR Population-Based Planning[Title/Abstract] OR Planning, Population-Based[Title/Abstract] OR Plannings, Population-Based[Title/Abstract] OR Population Based Planning[Title/Abstract] OR Population-Based Plannings[Title/Abstract] OR Community Health Systems[Title/Abstract] OR Community Health System[Title/Abstract] OR Health System, Community[Title/Abstract] OR Health Systems, Community[Title/Abstract] OR System, Community Health[Title/Abstract] OR Systems, Community Health[Title/Abstract] OR Therapeutic Community[Title/Abstract] OR Community, Therapeutic[Title/Abstract] OR Communities, Therapeutic[Title/Abstract] OR Therapeutic Communities[Title/Abstract] OR Community Medicine[Title/Abstract] OR Medicine, Community[Title/Abstract] OR Community Health Services[Title/Abstract] OR Community Health Service[Title/Abstract] OR Health Service, Community[Title/Abstract] OR Service, Community Health[Title/Abstract] OR Services, Community Health[Title/Abstract] OR Health Services, Community[Title/Abstract] OR Community Health Care[Title/Abstract] OR Care, Community Health[Title/Abstract] OR Health Care, Community[Title/Abstract] OR Community Healthcare[Title/Abstract] OR Community Healthcares[Title/Abstract] OR Healthcare, Community[Title/Abstract] OR Healthcares, Community[Title/Abstract] OR Community Health Centers[Title/Abstract] OR Center, Community Health[Title/Abstract] OR Centers, Community Health[Title/Abstract] OR Community Health Center[Title/Abstract] OR Health Center, Community[Title/Abstract] OR Health Centers, Community[Title/Abstract] OR Satellite Centers[Title/Abstract] OR Center, Satellite[Title/Abstract] OR Centers, Satellite[Title/Abstract] OR Satellite Center[Title/Abstract] OR Neighborhood Health Centers[Title/Abstract] OR Center, Neighborhood Health[Title/Abstract] OR Centers, Neighborhood Health[Title/Abstract] OR Health Center, Neighborhood[Title/Abstract] OR Health Centers, Neighborhood[Title/Abstract] OR Neighborhood Health Center[Title/Abstract] OR Community Health Nursing[Title/Abstract] OR Nursing, Community Health[Title/Abstract] OR Public Health[Title/Abstract] OR Health, Public[Title/Abstract] OR Community Health[Title/Abstract] OR Health, Community[Title/Abstract] OR Environment, Preventive Medicine[Title/Abstract] AND Residence Characteristics[Title/Abstract] OR Characteristic, Residence[Title/Abstract] AND Characteristics, Residence[Title/Abstract] OR Residence Characteristic[Title/Abstract] OR Domicile[Title/Abstract] OR Domiciles[Title/Abstract] OR Residential Selection[Title/Abstract] OR Residential Selections[Title/Abstract] OR Selection, Residential[Title/Abstract] OR Selections, Residential[Title/Abstract] OR Neighborhood[Title/Abstract] OR Neighborhoods[Title/Abstract] OR Place of Birth[Title/Abstract] OR Birth Place[Title/Abstract] OR Community[Title/Abstract] OR Communities[Title/Abstract] OR Living Arrangements[Title/Abstract] OR Arrangement, Living[Title/Abstract] OR Arrangements, Living[Title/Abstract] OR Living Arrangement[Title/Abstract] | 731597 |
| #9 | #7 OR #8 | 734238 |
| #10 | #3 AND #6 AND #9 | 1174 |

**Additional Table 2**

Full Search Queries Used in Web of Science (Updated October 17, 2022)

| Search | Query | Results |
| --- | --- | --- |
| #1 | TS=(Diabetes Mellitus, Type 2 OR Diabetes Mellitus, Noninsulin-Dependent OR Diabetes Mellitus, Ketosis-Resistant OR Diabetes Mellitus, Ketosis Resistant OR Ketosis-Resistant Diabetes Mellitus OR Diabetes Mellitus, Non Insulin Dependent OR Diabetes Mellitus, Non-Insulin-Dependent OR Non-Insulin-Dependent Diabetes Mellitus OR Diabetes Mellitus, Stable OR Stable Diabetes Mellitus OR Diabetes Mellitus, Type II OR NIDDM OR Diabetes Mellitus, Noninsulin Dependent OR Diabetes Mellitus, Maturity-Onset OR Diabetes Mellitus, Maturity Onset OR Maturity-Onset Diabetes Mellitus OR Maturity Onset Diabetes Mellitus OR Type 2 Diabetes Mellitus OR Noninsulin-Dependent Diabetes Mellitus OR Noninsulin Dependent Diabetes Mellitus OR Maturity-Onset Diabetes OR Diabetes, Maturity-Onset OR Maturity Onset Diabetes OR Type 2 Diabetes OR Diabetes, Type 2 OR Diabetes Mellitus, Adult-Onset OR Adult-Onset Diabetes Mellitus OR Diabetes Mellitus, Adult Onset) | 256570 |
| #2 | TS=(Exercise OR Exercises OR Physical Activity OR Activities, Physical OR Activity, Physical OR Physical Activities OR Exercise, Physical OR Exercises, Physical OR Physical Exercise OR Physical Exercises OR Exercise, Acute OR Exercises, Acute OR Exercise Training OR Exercise Trainings OR Training, Exercise OR Trainings, Exercise) | 848776 |
| #3 | TS=(Community health planing OR Community Health Plannings OR Health Planning, Community OR Health Plannings, Community OR Planning, Community Health OR Plannings, Community Health OR Population-Based Planning OR Planning, Population-Based OR Plannings, Population-Based OR Population Based Planning OR Population-Based Plannings OR Community Health Systems OR Community Health System OR Health System, Community OR Health Systems, Community OR System, Community Health OR Systems, Community Health OR Therapeutic Community OR Community, Therapeutic OR Communities, Therapeutic OR Therapeutic Communities OR Community Medicine OR Medicine, Community OR Community Health Services OR Community Health Service OR Health Service, Community OR Service, Community Health OR Services, Community Health OR Health Services, Community OR Community Health Care OR Care, Community Health OR Health Care, Community OR Community Healthcare OR Community Healthcares OR Healthcare, Community OR Healthcares, Community OR Community Health Centers OR Center, Community Health OR Centers, Community Health OR Community Health Center OR Health Center, Community OR Health Centers, Community OR Satellite Centers OR Center, Satellite OR Centers, Satellite OR Satellite Center OR Neighborhood Health Centers OR Center, Neighborhood Health OR Centers, Neighborhood Health OR Health Center, Neighborhood OR Health Centers, Neighborhood OR Neighborhood Health Center OR Community Health Nursing OR Nursing, Community Health OR Public Health OR Health, Public OR Community Health OR Health, Community OR Environment, Preventive Medicine and Public Health OR Environment, Preventive Medicine and Public Health OR Residence Characteristics OR Characteristic, Residence OR Characteristics, Residence OR Residence Characteristic OR Domicile OR Domiciles OR Residential Selection OR Residential Selections OR Selection, Residential OR Selections, Residential OR Neighborhood OR Neighborhoods OR Place of Birth OR Birth Place OR Community OR Communities OR Living Arrangements OR Arrangement, Living OR Arrangements, Living OR Living Arrangement) | 2626752 |
| #4 | #1 AND #2 AND #3 | 3784 |

**Additional Table 3**

Full Search Queries Used in The Cochrane Library (Updated October 17, 2022)

| Search | Query | Results |
| --- | --- | --- |
| #1 | Diabetes Mellitus, Type 2 | 63044 |
| #2 | (Diabetes Mellitus, Noninsulin-Dependent):ab,ti,kw OR (Diabetes Mellitus, Ketosis-Resistant):ab,ti,kw OR (Diabetes Mellitus, Ketosis Resistant):ab,ti,kw OR (Ketosis-Resistant Diabetes Mellitus):ab,ti,kw OR (Diabetes Mellitus, Non Insulin Dependent):ab,ti,kw OR (Diabetes Mellitus, Non-Insulin-Dependent):ab,ti,kw OR (Non-Insulin-Dependent Diabetes Mellitus):ab,ti,kw OR (Diabetes Mellitus, Stable):ab,ti,kw OR (Stable Diabetes Mellitus):ab,ti,kw OR (Diabetes Mellitus, Type II):ab,ti,kw OR (NIDDM):ab,ti,kw OR (Diabetes Mellitus, Noninsulin Dependent):ab,ti,kw OR (Diabetes Mellitus, Maturity-Onset):ab,ti,kw OR (Diabetes Mellitus, Maturity Onset):ab,ti,kw OR (Maturity-Onset Diabetes Mellitus):ab,ti,kw OR (Maturity Onset Diabetes Mellitus):ab,ti,kw OR (Type 2 Diabetes Mellitus):ab,ti,kw OR (Noninsulin-Dependent Diabetes Mellitus):ab,ti,kw OR (Noninsulin Dependent Diabetes Mellitus):ab,ti,kw OR (Maturity-Onset Diabetes):ab,ti,kw OR (Diabetes, Maturity-Onset):ab,ti,kw OR (Maturity Onset Diabetes):ab,ti,kw OR (Type 2 Diabetes):ab,ti,kw OR (Diabetes, Type 2):ab,ti,kw OR (Diabetes Mellitus, Adult-Onset):ab,ti,kw OR (Adult-Onset Diabetes Mellitus):ab,ti,kw OR (Diabetes Mellitus, Adult Onset):ab,ti,kw | 57687 |
| #3 | #1 OR #2 | 71889 |
| #4 | Exercise | 119088 |
| #5 | (Exercises OR Physical Activity OR Activities, Physical OR Activity, Physical OR Physical Activities OR Exercise, Physical OR Exercises, Physical OR Physical Exercise OR Physical Exercises OR Exercise, Acute OR Exercises, Acute OR Exercise Training OR Exercise Trainings OR Training, Exercise OR Trainings, Exercise) | 115558 |
| #6 | #4 OR #5 | 157418 |
| #7 | Community health planing | 36 |
| #8 | (Community health planing):ab,ti,kw OR (Community Health Plannings):ab,ti,kw OR (Health Planning, Community):ab,ti,kw OR (Health Plannings, Community):ab,ti,kw OR (Planning, Community Health):ab,ti,kw OR (Plannings, Community Health):ab,ti,kw OR (Population-Based Planning):ab,ti,kw OR (Planning, Population-Based):ab,ti,kw OR (Plannings, Population-Based):ab,ti,kw OR (Population Based Planning):ab,ti,kw OR (Population-Based Plannings):ab,ti,kw OR (Community Health Systems):ab,ti,kw OR (Community Health System):ab,ti,kw OR (Health System, Community):ab,ti,kw OR (Health Systems, Community):ab,ti,kw OR (System, Community Health):ab,ti,kw OR (Systems, Community Health):ab,ti,kw OR (Therapeutic Community):ab,ti,kw OR (Community, Therapeutic):ab,ti,kw OR (Communities, Therapeutic):ab,ti,kw OR (Therapeutic Communities):ab,ti,kw OR (Community Medicine):ab,ti,kw OR (Medicine, Community):ab,ti,kw OR (Community Health Services):ab,ti,kw OR (Community Health Service):ab,ti,kw OR (Health Service, Community):ab,ti,kw OR (Service, Community Health):ab,ti,kw OR (Services, Community Health):ab,ti,kw OR (Health Services, Community):ab,ti,kw OR (Community Health Care):ab,ti,kw OR (Care, Community Health):ab,ti,kw OR (Health Care, Community):ab,ti,kw OR (Community Healthcare):ab,ti,kw OR (Community Healthcares):ab,ti,kw OR (Healthcare, Community):ab,ti,kw OR (Healthcares, Community):ab,ti,kw OR (Community Health Centers):ab,ti,kw OR (Center, Community Health):ab,ti,kw OR (Centers, Community Health):ab,ti,kw OR (Community Health Center):ab,ti,kw OR (Health Center, Community):ab,ti,kw OR (Health Centers, Community):ab,ti,kw OR (Satellite Centers):ab,ti,kw OR (Center, Satellite):ab,ti,kw OR (Centers, Satellite):ab,ti,kw OR (Satellite Center):ab,ti,kw OR (Neighborhood Health Centers):ab,ti,kw OR (Center, Neighborhood Health):ab,ti,kw OR (Centers, Neighborhood Health):ab,ti,kw OR (Health Center, Neighborhood):ab,ti,kw OR (Health Centers, Neighborhood):ab,ti,kw OR (Neighborhood Health Center):ab,ti,kw OR (Community Health Nursing):ab,ti,kw OR (Nursing, Community Health):ab,ti,kw OR (Public Health):ab,ti,kw OR (Health, Public):ab,ti,kw OR (Community Health):ab,ti,kw OR (Health, Community):ab,ti,kw OR (Environment, Preventive Medicine and Public Health):ab,ti,kw OR (Environment, Preventive Medicine and Public Health):ab,ti,kw OR (Residence Characteristics):ab,ti,kw OR (Characteristic, Residence):ab,ti,kw OR (Characteristics, Residence):ab,ti,kw OR (Residence Characteristic):ab,ti,kw OR (Domicile):ab,ti,kw OR (Domiciles):ab,ti,kw OR (Residential Selection):ab,ti,kw OR (Residential Selections):ab,ti,kw OR (Selection, Residential):ab,ti,kw OR (Selections, Residential):ab,ti,kw OR (Neighborhood):ab,ti,kw OR (Neighborhoods):ab,ti,kw OR (Place of Birth):ab,ti,kw OR (Birth Place):ab,ti,kw OR (Community):ab,ti,kw OR (Communities):ab,ti,kw OR (Living Arrangements):ab,ti,kw OR (Arrangement, Living):ab,ti,kw OR (Arrangements, Living):ab,ti,kw OR (Living Arrangement):ab,ti,kw | 74542 |
| #9 | #7 OR #8 | 74556 |
| #10 | #3 AND #6 AND #9 | 1243 |

**Additional Table 4**

Full Search Queries Used in Embase (Updated October 17, 2022)

| Search | Query | Results |
| --- | --- | --- |
| #1 | ('diabetes'/exp OR diabetes) AND mellitus, AND type AND ('2'/exp OR 2) | 389700 |
| #2 | 'diabetes mellitus, noninsulin-dependent':ab,ti OR 'diabetes mellitus, ketosis-resistant':ab,ti OR 'diabetes mellitus, ketosis resistant':ab,ti OR 'ketosis-resistant diabetes mellitus':ab,ti OR 'diabetes mellitus, non insulin dependent':ab,ti OR 'diabetes mellitus, non-insulin-dependent':ab,ti OR 'non-insulin-dependent diabetes mellitus':ab,ti OR 'diabetes mellitus, stable':ab,ti OR 'stable diabetes mellitus':ab,ti OR 'diabetes mellitus, type ii':ab,ti OR 'niddm':ab,ti OR 'diabetes mellitus, noninsulin dependent':ab,ti OR 'diabetes mellitus, maturity-onset':ab,ti OR 'diabetes mellitus, maturity onset':ab,ti OR 'maturity-onset diabetes mellitus':ab,ti OR 'maturity onset diabetes mellitus':ab,ti OR 'type 2 diabetes mellitus':ab,ti OR 'noninsulin-dependent diabetes mellitus':ab,ti OR 'noninsulin dependent diabetes mellitus':ab,ti OR 'maturity-onset diabetes':ab,ti OR 'diabetes, maturity-onset':ab,ti OR 'maturity onset diabetes':ab,ti OR 'type 2 diabetes':ab,ti OR 'diabetes, type 2':ab,ti OR 'diabetes mellitus, adult-onset':ab,ti OR 'adult-onset diabetes mellitus':ab,ti OR 'diabetes mellitus, adult onset':ab,ti | 248224 |
| #3 | #1 OR #2 | 417769 |
| #4 | exercise | 654906 |
| #5 | 'Exercises':ab,ti OR 'Physical Activity':ab,ti OR 'Activities, Physical':ab,ti OR 'Activity, Physical':ab,ti OR 'Physical Activities':ab,ti OR 'Exercise, Physical':ab,ti OR 'Exercises, Physical':ab,ti OR 'Physical Exercise':ab,ti OR 'Physical Exercises':ab,ti OR 'Exercise, Acute':ab,ti OR 'Exercises, Acute':ab,ti OR 'Exercise Training':ab,ti OR 'Exercise Trainings':ab,ti OR 'Training, Exercise':ab,ti OR 'Trainings, Exercise':ab,ti | 288170 |
| #6 | #4 OR #5 | 787072 |
| #7 | community AND health AND planing | 90 |
| #8 | 'community health plannings':ab,ti OR 'health planning, community':ab,ti OR 'health plannings, community':ab,ti OR 'planning, community health':ab,ti OR 'plannings, community health':ab,ti OR 'population-based planning':ab,ti OR 'planning, population-based':ab,ti OR 'plannings, population-based':ab,ti OR 'population based planning':ab,ti OR 'population-based plannings':ab,ti OR 'community health systems':ab,ti OR 'community health system':ab,ti OR 'health system, community':ab,ti OR 'health systems, community':ab,ti OR 'system, community health':ab,ti OR 'systems, community health':ab,ti OR 'therapeutic community':ab,ti OR 'community, therapeutic':ab,ti OR 'communities, therapeutic':ab,ti OR 'therapeutic communities':ab,ti OR 'community medicine':ab,ti OR 'medicine, community':ab,ti OR 'community health services':ab,ti OR 'community health service':ab,ti OR 'health service, community':ab,ti OR 'service, community health':ab,ti OR 'services, community health':ab,ti OR 'health services, community':ab,ti OR 'community health care':ab,ti OR 'care, community health':ab,ti OR 'health care, community':ab,ti OR 'community healthcare':ab,ti OR 'community healthcares':ab,ti OR 'healthcare, community':ab,ti OR 'healthcares, community':ab,ti OR 'community health centers':ab,ti OR 'center, community health':ab,ti OR 'centers, community health':ab,ti OR 'community health center':ab,ti OR 'health center, community':ab,ti OR 'health centers, community':ab,ti OR 'satellite centers':ab,ti OR 'center, satellite':ab,ti OR 'centers, satellite':ab,ti OR 'satellite center':ab,ti OR 'neighborhood health centers':ab,ti OR 'center, neighborhood health':ab,ti OR 'centers, neighborhood health':ab,ti OR 'health center, neighborhood':ab,ti OR 'health centers, neighborhood':ab,ti OR 'neighborhood health center':ab,ti OR 'community health nursing':ab,ti OR 'nursing, community health':ab,ti OR 'public health':ab,ti OR 'health, public':ab,ti OR 'community health':ab,ti OR 'health, community':ab,ti OR 'environment, preventive medicine and public health':ab,ti OR 'residence characteristics':ab,ti OR 'characteristic, residence':ab,ti OR 'characteristics, residence':ab,ti OR 'residence characteristic':ab,ti OR 'domicile':ab,ti OR 'domiciles':ab,ti OR 'residential selection':ab,ti OR 'residential selections':ab,ti OR 'selection, residential':ab,ti OR 'selections, residential':ab,ti OR 'neighborhood':ab,ti OR 'neighborhoods':ab,ti OR 'place of birth':ab,ti OR 'birth place':ab,ti OR 'community':ab,ti OR 'communities':ab,ti OR 'living arrangements':ab,ti OR 'arrangement, living':ab,ti OR 'arrangements, living':ab,ti OR 'living arrangement':ab,ti | 1192620 |
| #9 | #7 OR #8 | 1192665 |
| #10 | #3 AND #6 AND #9 | 2689 |
